# Impact of systolic blood pressure limits on the diagnostic value of triage algorithms

**DOI:** 10.1186/s13049-017-0461-2

**Published:** 2017-12-04

**Authors:** Tobias Neidel, Nicolas Salvador, Axel R. Heller

**Affiliations:** Department of Anesthesiology and Critical Care Medicine, Medical Faculty Carl Gustav Carus, TU-Dresden, Fetscherstrasse 74, D-01307 Dresden, Germany

**Keywords:** Triage algorithms, Diagnostic value, Palpable pulse, SBP-limits, Major incidents

## Abstract

**Background:**

Major incidents are characterized by a lack of resources compared to an overwhelming number of casualties, requiring a prioritization of medical treatment. Triage algorithms are an essential tool for prioritizing the urgency of treatment for patients, but the evidence to support one over another is very limited. We determined the influence of blood pressure limits on the diagnostic value of triage algorithms, considering if pulse should be palpated centrally or peripherally.

**Methods:**

We used a database representing 500 consecutive HEMS patients. Each patient was allocated a triage category (T1/red, T2/yellow, T3/green) by a group of experienced doctors in disaster medicine, independent of any algorithm. mSTaRT, ASAV, Field Triage Score (FTS), Care Flight (CF), “Model Bavaria” and two Norwegian algorithms (Nor and TAS), all containing the question “Pulse palpable?”, were translated into Excel commands, calculating the triage category for each patient automatically. We used 5 blood pressure limits ranging from 130 to 60 mmHg to determine palpable pulse. The resulting triage categories were analyzed with respect to sensitivity, specificity and Youden Index (J) separately for trauma and non-trauma patients, and for all patients combined.

**Results:**

For the entire population of patients within all triage algorithms the Youden Index (J) was highest for T1 (J between 0,14 and 0,62). Combining trauma and non-trauma patients, the highest J was obtained by ASAV (J = 0,62 at 60 mmHg). ASAV scored the highest within trauma patients (J = 0,87 at 60 mmHg), whereas Model Bavaria (J = 0,54 at 80 mmHg) reached highest amongst non-trauma patients. FTS performed worst for all patients (J = 0,14 at 60 mmHg), showing a lower score for trauma patients (J = 0,0 at 60 mmHg). Change of blood pressure limits resulted in different diagnostic values of all algorithms.

**Discussion:**

We demonstrate that differing blood pressure limits have a remarkable impact on diagnostic values of triage algorithms. Further research is needed to determine the lowest blood pressure value that is possible to palpate at a peripheral artery compared to a central artery.

**Conclusion:**

As a consequence, it might be important in which location pulses are palpated according to the algorithm at hand during triage of patients.

## Background

Mass casualty incidents frequently force the established emergency systems to work on the edge of their capabilities, or even beyond. The Department of Health’s Strategic National Guidance to the NHS for Major Incident Emergency Planning (2005) defines a major incident as any occurrence that presents a serious threat to the health of the community, disruption to the service or causes such numbers or types of casualties as to require special arrangements to be implemented by hospitals, ambulance trusts or primary care organizations [1]. Due to this circumstance, the medical treatment has to be prioritized to reach the best outcome for the whole cohort of patients. This requires a fast and brief assessment of the injuries, not spending too much time on the individual patient. To make this possible, several triage procedures were developed over the years, originating out of a military focus and now regularly used in the civilian emergency medicine [2]. Still, the key task is to provide maximum benefit to the most people [3]. Usually the patients are classified into one of three categories (T1-T3) and deceased people, referred to as “red, yellow, green, and black” accordingly, with T1 representing the highest urgency and T3 the lowest. There is one additional category existing (T4 resp. “blue”), for cases for which the available resources are overwhelmed. In most countries, this category is only allowed to be used if the commanding medical officer on scene has decided that it is necessary due to a massive lack of resources making it impossible to treat all T1 patients. Therefore, this category should usually not be allocated by non-physicians [4].

One key issue for the triage procedure is to find the most urgent patients in a fast but also very accurate way. Categorizing too many casualties into T1 (over-triage) may cause an allocation of medical resources to people who are not as much in the need of it as more severely injured ones. On the other hand, not classifying urgent patients into the highest group (under-triage), may delay their treatment and cause a worse outcome or even death. Therefore, triage algorithms should have the highest possible sensitivity and specificity regarding classification into T1 (category red). Although many algorithms have been developed and are used in the field, the evidence to support one over another is very limited [2, 5]. Heller et al. has already published the underlying database and basic method of our project. The results showed that the triage algorithms mSTaRT and ASAV might perform with the highest sensitivity and specificity in the field, whereas PRIOR might cause an overtriage of not severely injured victims. Heller et al. also reported that all triage algorithms generally work better for trauma compared to non-trauma patients [5].

In the first editions of Advanced Trauma Life Support (ATLS) course manual [6], it was stated that the presence of carotid (> 60 mmHg), femoral (> 70 mmHg), and radial pulse (> 80 mmHg) would correlate to a certain systolic blood pressure (SBP) in hypotensive trauma patients. Following the editorial by Poulton [7] stating a lack of correlation of palpable pulses with SBP, this doctrine was withdrawn from ATLS and corresponding course manuals. Considering this uncertainty, triage algorithms using the presence of pulses for decision-making on patient allocation to triage categories apparently vary in their diagnostic quality depending on the true SBP. To address this issue, we investigated the diagnostic value of several frequently used triage algorithms, with a special focus on the influence of changing SBP limits, connecting the question if pulse should be palpated at a peripheral or a central artery.

## Methods

After institutional ethical review board approval by the Medical Faculty of the Technical University of Dresden (EK DD 270 06 2015) we used a database representing 500 consecutive patients treated by Helicopter Emergency Medical Service (HEMS). Both the electronic emergency documentation and the hand-written documentations were used for the database. Eight patients were already dead when arriving on the scene. These were excluded for this study, so that 492 patients were used for further evaluation. Each patient was allocated a triage category (T1/red, T2/yellow, T3/green) by a group of experienced doctors in disaster medicine, independent of any algorithm merely regarding the definition of the triage categories according to the 6th Triage-Consensus conference of the German Federal Office of Civil Protection and Disaster Assistance as shown in Table 1 [4].

**Table 1:**
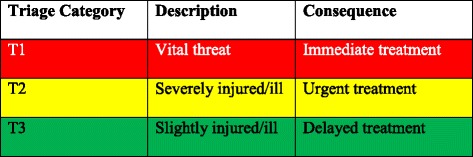
Description of triage categories

This procedure has already been published in more detail by our group [5]. The triage algorithms “modified Simple Triage and Rapid Treatment” (mSTaRT, Version 2013), Amberg-Schwandorf-Algorithmus (ASAV), Field Triage Score (FTS), Care Flight (CF), “Model Bavaria” (based on mSTaRT) [8–11] and two Norwegian algorithms, one used by personnel without further medical education (TAS) [12] and one used by medical professionals (in this Paper called “Nor”) [13], both based on the algorithm Triage Sieve by MIMMS, were translated into Microsoft Excel commands. The exact Excel commands are available from the corresponding author upon request. Every algorithm requests at a certain position whether or not pulse is palpable. In order to decide yes or no, we defined the limits for the systolic blood pressure being ≥ 130 mmHg, ≥ 110 mmHg, ≥ 100 mmHg, ≥ 80 mmHg or ≥ 60 mmHg [14]. This made it possible to calculate the triage categories for every patient automatically depending on each defined limit.

The 2013 version of mSTaRT contains a second triage procedure for patients who were classified as “green” (T3). As there were no clearly defined criteria for this second triage, we decided to test the initially “green” classified patients for the criteria of category “red” in the same algorithm. Patients who had no “red”-criterion during secondary survey were left in T3. All other patients were not allowed to stay in this category and were passed on to the next steps of the algorithm. Both mSTaRT 2013 and “Model Bavaria” ask if the patient suffers from an “Inhalation Trauma with Stridor”. No patient of our cohort showed this trauma in the documentation, so we decided to skip this question in the algorithms.

As the Field Triage Score can reach the values 0, 1 and 2, we calculated +1 to be comparable to the triage categories I to III.

Statistical analyses were done with respect to sensitivity (SE), specificity (SP), positive predictive value (PPV), negative predictive value (NPV), positive/negative Likelihood Ratio (LR+, LR-) and the Youden Index (J), representing a marker that combines sensitivity and specificity and ranging from −1 to +1 [15], making the results of the Receiver Operating Characteristics (ROC) easier to compare.

## Results

A total of 492 patients were included in the study - 212 female and 380 male. One hundred ninety four suffered from a traumatic emergency with a mean age of 49 (±2) years and 298 suffered from a non-trauma emergency with a mean age of 65 years (±1). The distribution of patients within the different blood pressure limits is shown in Table 2.

**Table 2:** Distribution of patients over the different blood pressure limits

Systolic SBP-Limit [mmHg]	≥130	≥110	≥100	≥80	≥60	<60
Total n Patients	320	406	449	476	483	9
n Trauma Patients	127	167	182	193	194	0
n Non-trauma Patients	193	239	267	283	289	9

**Fig. 1:**
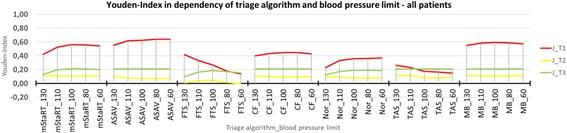
Youden-Index in dependency of triage algorithm and blood pressure limit – all patients. Youden Index is shown for all triage algorithms and blood pressure limits each. Trauma and non-trauma patients are combined

Figure 1 shows that the Youden Index for T1 (red) is higher compared to T2 (yellow) or T3 (green). Further, changing the blood pressure limit influences the diagnostic value, with some algorithms (FTS and TAS) preferring higher limits around 130 mmHg and some (mSTaRT 2013, ASAV and Nor) preferring lower limits. CF and “Model Bavaria” were least influenced. The following results are concentrating on triage category 1.

### Whole patient cohort

**Fig. 2:**
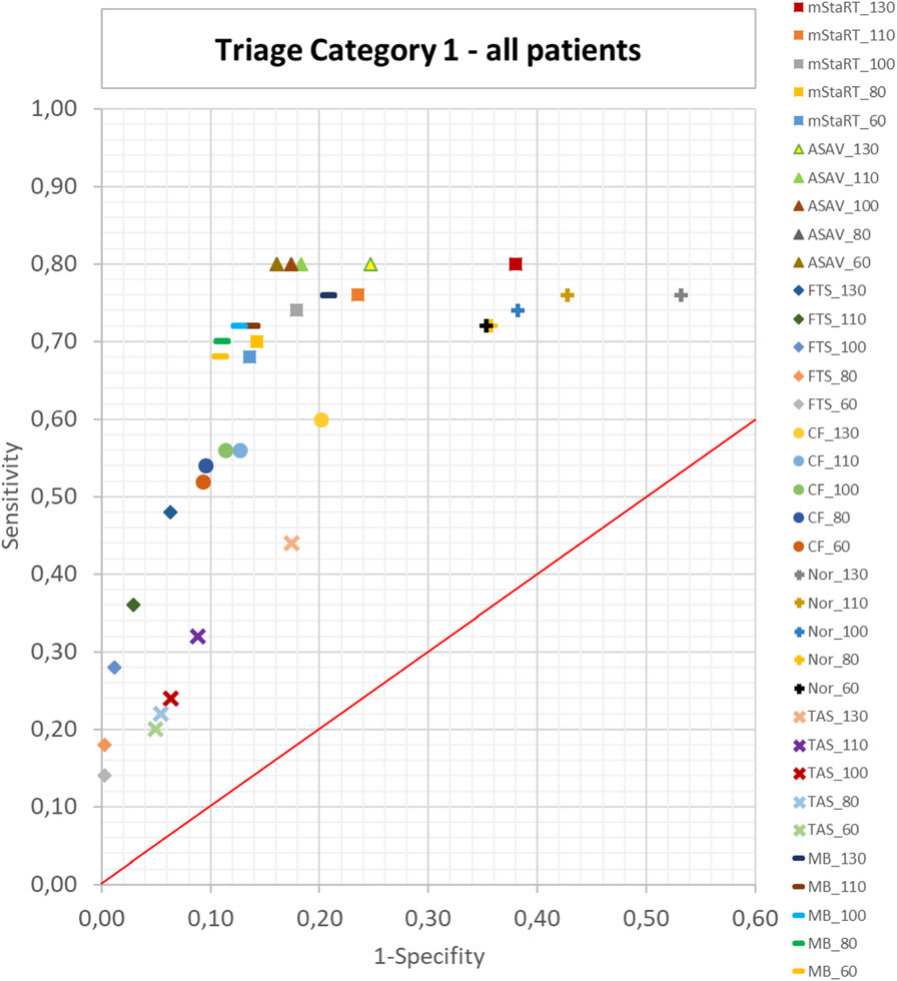
Triage Category 1 – all patients. Sensitivity and 1-Specificity is shown for every algorithm and blood pressure limit each. Results for T1 are shown for trauma and non-trauma patients combined. The red line represents a Youden Index of 0. Results in the upper right corner are classifying tendentially too many patients in T1 and in the lower left corner too few patients in T1

The results for the whole cohort of patients are shown regarding the Youden Indices in Fig. 1 and regarding the sensitivity and specificity in Fig. 2. ASAV reached the highest Youden Indices when combining trauma and non-trauma patients with the best value of 0,64. Model Bavaria and mSTaRT are following with values of 0,59 and 0,56. ASAV showed a clear tendency to perform better with lower blood pressure limits, whereas mSTaRT and Model Bavaria reached the best Indices at limits around 100 mmHg. The Care Flight algorithm also scored its highest J of 0,45 at 100 mmHg but performed, in general, worse than the previous 3 algorithms. “Nor” preferred lower systolic blood pressure limits and scored a J of 0,37 at 60 mmHg. FTS and TAS both reached best results of 0,42 and 0,27 at 130 mmHg.

Figure 2 shows, that the algorithms ASAV, Model Bavaria and mSTaRT are the most balanced regarding over- or undertriage for triage category one. However, mSTaRT tends to overtriage patients, when using high blood pressure limits (130 mmHg). Nor generally tends to result in overtriage, but performs better when using lower limits (60 mmHg). On the contrary, TAS and FTS tend to result in undertriage, but perform better with high blood pressure limits (130 mmHg).

When analyzing the second round of triage of category three within mSTaRT it was seen that 12 of 272 initially as “green” classified patients (4,4%) were upgraded to the category “red” using the SBP-limit 60 mmHg, with an increasing number of patients up to 78 (28,7%) using the SBP-limit 130 mmHg.

### Trauma patients

**Fig. 3:**
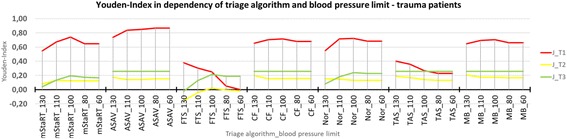
Youden-Index in dependency of triage algorithm and blood pressure limit – trauma patients. Youden Index is shown for all triage algorithms and blood pressure limits each. Only trauma patients are shown

**Fig. 4:**
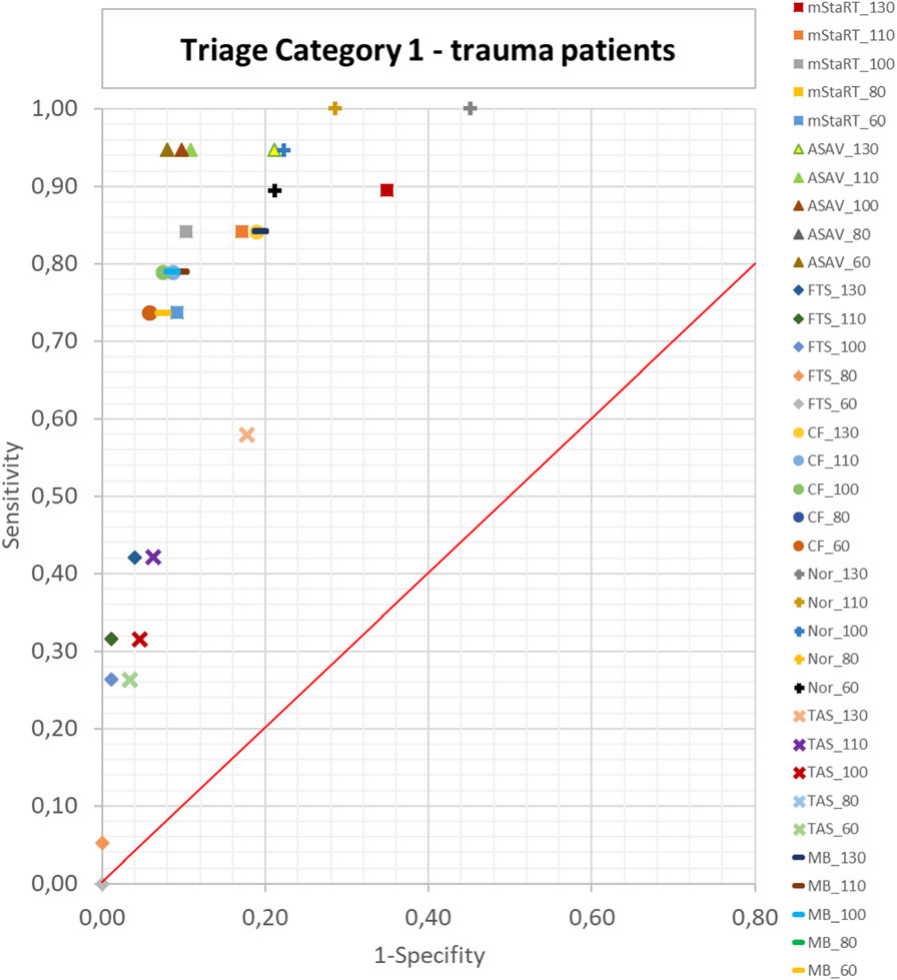
Triage Category 1 – trauma patients. Sensitivity and 1-Specificity is shown for every algorithm and blood pressure limit each. Results for T1 are shown for trauma patients only. The red line represents a Youden Index of 0. Results in the upper right corner are classifying tendentially too many patients in T1 and in the lower left corner too less patients in T1

Results for trauma patients are shown in Figs. 3 and 4. Six out of 7 algorithms showed a better performance for trauma patients compared to the whole patient cohort. Only the Field Triage Score performed worse. ASAV reached J = 0,87, which was the highest Index with the best results for the limits 80 and 60 mmHg. mSTaRT followed with J = 0,74 at 100 mmHg. CF, Nor and Model Bavaria also reached their highest J at 100 mmHg with values of 0,72 (CF, Nor) and 0,70 (Model Bavaria). TAS and FTS reached their highest Indices of 0,40 and 0,38 at 130 mmHg. FTS scored at the limit of 60 mmHg the lowest Youden Index with 0,0 representing the lowest value of the whole study.

Figure 4 shows that the behaviour regarding over- or undertriage remains the same, as was seen for the whole cohort (Fig. 2). ASAV, mSTaRT and Model Bavaria were again the most balanced algorithms, with mSTaRT tending to result in overtriage when using the high blood pressure limits. Nor also tended towards overtriage but performed better with low SBP-limits. FTS and TAS tended to undertriage with better performance when using low blood pressure limits.

### Non-trauma patients

**Fig. 5:**
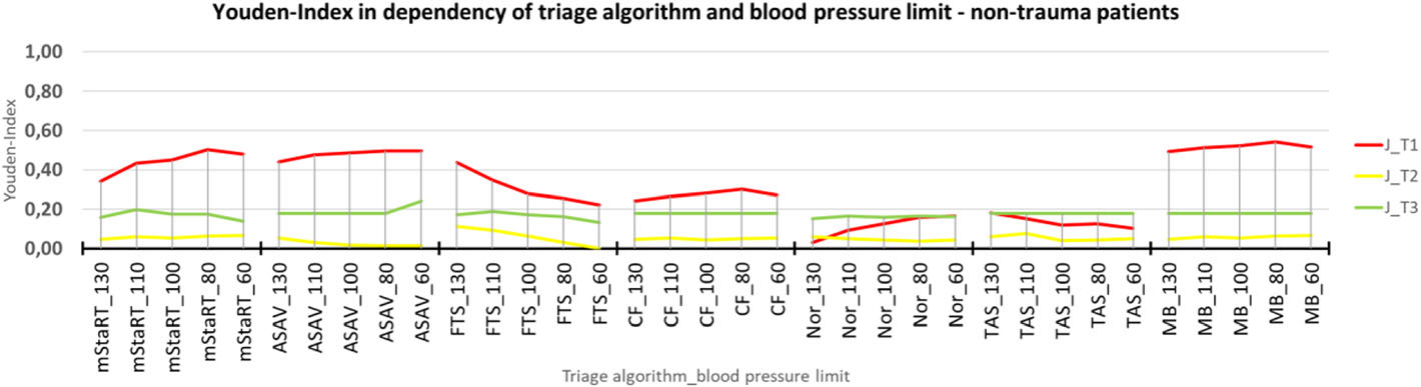
Youden-Index in dependency of triage algorithm and blood pressure limit – non-trauma patients. Youden Index is shown for all triage algorithms and blood pressure limits each. Only non-trauma patients are shown

**Fig. 6:**
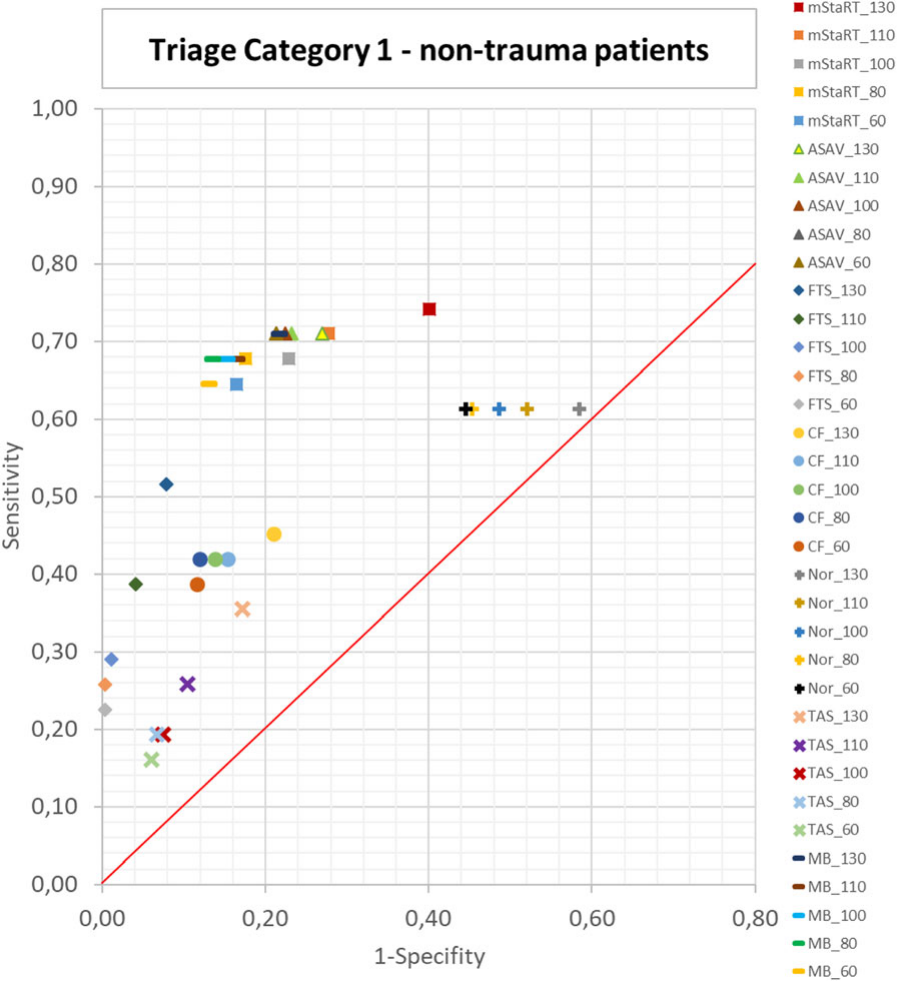
Triage Category 1 – non-trauma patients. Sensitivity and 1-Specificity is shown for every algorithm and blood pressure limit each. Results for T1 are shown for non-trauma patients only. The red line represents a Youden Index of 0. Results in the upper right corner are classifying tendentially too many patients in T1 and in the lower left corner too less patients in T1

Results for the non-trauma patients are shown in Figs. 5 and 6. The performance of almost all algorithms is worse when used for non-trauma patients. Only the Field Triage Score performed slightly better, compared to the results for the whole cohort. In this case, Model Bavaria scored the highest Youden Index with 0,54 at 80 mmHg, followed by mSTaRT and ASAV each with 0,50 at 80 mmHg. FTS reached with J = 0,44 at 130 mmHg a higher Youden Index for non-trauma patients, than for trauma patients. CF resulted in a J of 0,3 at 80 mmHgh. Nor and TAS performed worst for non-trauma patients with J = 0,17 at 60 mmHg and J = 0,18 at 130 mmHg respectively.

Figure 6 shows that almost all algorithms behaved in a similar manner when used for non-trauma patients as for the whole cohort. Only the Care Flight algorithm showed a new tendency to result in undertriage.

## Discussion

As was expected based on clinical experience, our results clearly show that varying blood pressure limits affect the test quality of triage algorithms. The seemingly sound, early ATLS doctrine that the presence of carotid (> 60 mmHg), femoral (> 70 mmHg), and radial pulse (> 80 mmHg) would correlate with a certain systolic blood pressure (SBP) in hypotensive trauma patients [6] was never scientifically proven. One underpowered study, however, claimed high variability in the measurements and showed a certain degree of underestimating the real blood pressure [16] by the pulse status. Regarding Body-Mass-Index, pre-existing vascular diseases, or other sources of variability, at given blood pressure levels palpability of pulses may also be grossly affected. Recent studies tried to improve the evidence for estimating the blood pressure according to the palpation of pulse [14]. Regardless of whether or not the correlation between pulse and SBP is scientifically proven or not, the creators of triage algorithms included the idea that palpable pulse reflects a certain degree of SBP. What we learned from the present study is that the assumed SBP cutoff is important for the correct assignment of a certain patient to a triage category. One further factor for the patient assignment is the capability of on-scene health care providers to correctly check for the pulse at appropriate locations. Accordingly, this implies that the location of where the pulse is palpated, e.g. radial vs. carotid artery, is relevant for the proper performance of the algorithms.

Despite all underlying variability and poor data supporting the correlation between pulse status and SBP, this assumption was integral to the foundation of triage algorithms and the correlation still appears physiologically sound. Consequently, the location of pulse measurement is of significance for the predictive value of the algorithms. Within this context and under the physiological assumption, that pulse is still palpable at a central artery at such low values that it is not possible to palpate it at a peripheral artery, it may be advisable for the algorithms ASAV and Nor to check for pulses at a central artery because such algorithms scored the highest Youden Index at the lowest SBP-limits (Figs. 1, 3 and 5). In the cases of TAS and FTS, pulse should be checked at a peripheral artery as those algorithms are scoring their highest Youden Indices at the highest SBP-limits (Figs. 1, 3 and 5). When considering mSTaRT, “Model Bavaria” and Care Flight is it more difficult to offer recommendations, as the highest Youden Indices are scored for blood pressure limits somewhere in between 130 and 60 mmHg. However, these algorithms did show slightly worse performance for the limit 130 mmHg compared to 60 mmHg (Figs. 1, 3 and 5). Therefore, users of such algorithms should preferably check the central arteries.

For a population of patients similar to the cohort presenting in daily emergency services, the algorithms mSTaRT, “Model Bavaria” and ASAV showed the best performance regarding the scored Youden Indices (Fig. 1). Regarding mSTaRT or respectively “Model Bavaria” it is to be noticed, that a potentially high number of severely injured people can be missed in the first triage, when the one and only criterion for T3 (green) is the ability of a patient to walk [9]. The fact that this decision is made as the first step within the algorithm increases the risk of under-triage. In our study, this issue affected between 4,4% and 28,7% of all primarily “green” classified patients. If an algorithm like “Model Bavaria” is used, a second evaluation of the patients in T3 is absolutely necessary.

Taking into account that most of the patients during an acute major incident will present with traumatic injuries, the algorithms ASAV and mSTaRT seem to be most suitable for this situation. A surprisingly poor performance was observed in the Field Triage Score for this group of patients (Fig. 3), as it is supposed to be used for injured soldiers, who will mainly suffer from traumatic injuries during combat. In this regard FTS appears as a long term outcome predicting score [11] rather than a triage algorithm for defining actual patient demands. The currently used blood pressure limit of this algorithm is at 100 mmHg [11]. As shown, the FTS tends to provide poor performance when using low blood pressure limits (Figs. 1, 3 and 5). Thus, we recommend changing blood pressure limit to values around 110 or 130 mmHg and checking the pulse at peripheral artery, in order to increased diagnostic value. Due to its low sensitivity, however, it misses more than half of all severely injured. Therefore, it should be discussed if significant changes in the algorithm design need to be carried out to ensure that the use of FTS in combat situations is safe for affected soldiers. Every other algorithm, apart from FTS, performed better in trauma patients than in non-trauma patients. This observation is in line with a study on accuracy of primary diagnosis in the emergency room compared to the primary diagnosis at discharge -- which was considerably higher for trauma compared to non-trauma diagnoses [17].

Under conditions of increased caseload of non-trauma patients, the “Model Bavaria” should be considered alongside mSTaRT and ASAV, as it shows the best diagnostic quality for this specific group of patients (Fig. 5). Recent database analyses showed a considerably higher proportion of Mass Casualty Incidents with non-trauma patients [18]. This may also be a typical Medical Task Forces mission scenario, when medical disaster relief units are deployed to work for a longer period of time on the scene (e.g. weeks) [19].

It is interesting to see, that the two Norwegian algorithms [13] are somehow working opposite to one another. TAS is used by non-medical personnel and tends to classify too few patients as “red” (under-triage) (Figs. 2, 4 and 6). On the contrary, Nor, which is used by medical personal, tends to classify too many patients as “red” (over-triage) (Figs. 2, 4 and 6). This is particularly true for trauma patients. The reason for this may be that TAS is sorting out the “green” patients at the very beginning, while when using Nor, this category appears at the end of the algorithm. The later observation is also pertinent to the over-triaging German PRIOR algorithm, which also provides many diversions for reaching the red category, before green can finally be accessed [5]. Additionally, TAS performs better when pulse is checked at a peripheral artery and Nor performs better, when using a central artery (Figs. 1, 3 and 5). This is consistent with the expected medical skill-level of the people using those algorithms.

## Conclusion

We were able to demonstrate that changing blood pressure limits within triage algorithms affect their test quality. Further research needs to be carried out to determine the limits at which blood pressure the pulse can be checked at the different body locations and if other signs of circulation should be preferred in algorithms. In the light of the present data the position where to check the pulse must clearly be defined dependent on the used triage algorithm. Taking these steps could improve the accuracy of finding the “red” patients, first giving medical treatment to those who are in need of it most.

## Abbreviations

ASAV: Amberg-Schwandorf-Algorithm
ATLS: Advanced Trauma Life Support
BMI: Body-Mass-Index
CF: Care Flight
FTS: Field Triage Score
J: Youden Index
LR-: Negative Likelihood Ratio
LR+: Positive Likelihood Ratio
MB: Model Bavaria
mSTaRT: Modified Simple Triage and Rapid Treatment
NHS: National Health Service
Nor: Norwegian triage algorithm for medical personnel developed by Norwegian Air Ambulance Foundation
SBP: Systolic Blood Pressure
TAS: Triage algorithm for non-medical personnel developed by Norwegian Air Ambulance Foundation
